# Clinicians’ experiences with cancer patients living longer with incurable cancer: a focus group study in the Netherlands

**DOI:** 10.1017/S1463423622000500

**Published:** 2023-04-28

**Authors:** Hilde M. Buiting, Femke Botman, Lilly-Ann van der Velden, Linda Brom, Florien van Heest, Eva E. Bolt, Pieter de Mol, Ton Bakker

**Affiliations:** 1 Netherlands Cancer Institute, Antoni van Leeuwenhoek, Department of Medical Oncology, Head & Neck Surgery, and Thoracic Oncology, Amsterdam, the Netherlands; 2 University of Amsterdam, Amsterdam, the Netherlands; 3 O2PZ, Platform of Palliative Care, Amsterdam, the Netherlands; 4 Amsterdam UMC, VUmc, Department of Medical Oncology, Amsterdam, the Netherlands; 5 Netherlands Cancer Institute, Antoni van Leeuwenhoek, Department of Head and Neck Oncology, Amsterdam, the Netherlands; 6 Netherlands Comprehensive Cancer Organisation, Department of Research, Utrecht, the Netherlands; 7 GP Practice Schoonoord, Schoonoord, the Netherlands; 8 Amsterdam UMC, VUmc, Department of Public and Occupational Health, Amsterdam, the Netherlands; 9 Hospital Gelderse Vallei, Department of Medical Oncology, Ede, the Netherlands; 10 Science in Balance Foundation, Rotterdam, the Netherlands; 11 Rotterdam University of Applied Sciences, Rotterdam, the Netherlands

**Keywords:** cancer, chronic disease, decision-making, doctor–patient communication

## Abstract

**Aim:**

To explore (1) experiences of primary care physicians (PCPs) and oncological medical specialists about providing care to patients living longer with incurable cancer, and (2) their preferences concerning different care approaches (palliative support, psychological/survivorship care support).

**Background:**

At present, oncological medical specialists as well as PCPs are exploring how to improve and better tailor care to patients living longer with incurable cancer. Our previous study at the in-patient oncology unit showed that patients living longer with incurable cancer experience problems in how to deal with a prognosis that is insecure and fluctuating. To date, it could be argued that treating these patients can be done with a ‘palliative care’ or a ‘survivorship/psychosocial care’ approach. It is unknown what happens in actual medical practice.

**Methods:**

We performed multidisciplinary group meetings: 6 focus groups (3 homogenous groups with PCPs (*n* = 15) and 3 multidisciplinary groups (*n* = 17 PCPs and n = 6 medical specialists) across different parts of the Netherlands. Qualitative data were analysed with thematic analysis.

**Findings and conclusions:**

In the near future, PCPs will have an increasing number of patients living longer with incurable cancer. However, in a single PCP practice, the experience with incurable cancer patients remains low, partly because patients often prefer to stay in contact with their medical specialist. PCPs as well as medical specialists show concerns in how they can address this disease phase with the right care approach, including the appropriate label (e.g. palliative, chronic, etc.). They all preferred to be in contact early in the disease process, to be able to discuss and take care for the patients’ physical and psychological well-being. Medical specialists can have an important role by timely referring their patients to their PCPs. Moreover, the disease label ‘chronic’ can possibly assist patients to live their life in the best possible way.

## Introduction

Advances in oncology have resulted in an increased number of cancer survivors (Harley *et al*. 2015; Heins *et al*. 2015; IKNL, 2022). This has resulted in an increased life span for many patients with incurable cancer. Some forms of cancer (especially breast, prostate and colon cancer as well as haematological cancers) seem to slowly develop into ‘chronic’ diseases (Harley *et al*. 2015; Buiting *et al*. 2019). Up till now, it is to a great extent unknown how these patients should be approached and defined to serve them best (Schildmann *et al*. 2020).

It could be argued that in patients living longer with incurable cancer (e.g. > 1 year) two distinct care approaches could be applied: a palliative care approach and a survivorship/psychosocial care approach. A palliative care approach is aimed at improving the quality of life of patients with life-threatening illness and their families, without the aim of life-prolongation (Fadul *et al*. 2009; Thoonsen *et al*. 2016). During the provision of anti-cancer treatment (e.g. ‘standard oncology care’), this is usually integrated with elements of palliative care, from diagnosis of incurable cancer until death (Murray *et al*. 2005; Greer *et al*. 2012; Frick *et al*. 2017). Studies about early palliative care often encompass care for approximately one year; longer periods have not been studied. A survivorship care approach is a different care approach. According to Frick *et al* (among others), this approach also appeals to many patients living longer with incurable cancer (Frick *et al*. 2017). This approach focuses on quality of life as well as on survival and includes interventions aimed at optimal living (Starreveld *et al*. 2018). Both approaches could apply to care for patients living longer with incurable cancer, but the philosophies (and medical specialties) of both approaches differ.

At present, oncologists as well as primary care physicians (PCPs) are exploring how to improve care that is better tailored to patients living longer with incurable cancer. Our previous study at the in-patient oncology unit showed that patients living longer with incurable cancer experience problems in how to deal with a prognosis that is insecure (Buiting *et al*. 2019). In the Netherlands, almost everyone has a PCP and patients can consult a PCP free-of-charge (Dutch Patient Federation). The Dutch Cancer Society (KWF) and the Dutch Health Council already propagated 9 years ago that PCPs do have an important task in the care for patients living longer with incurable cancer (KWF 2011). It could be argued that patients living longer with incurable cancer could be (partially) followed by PCPs as well. Right now, there is no established framework about the right care approach for patients living longer with incurable cancer, as the care approach for incurable patients mainly focuses on end-stage/terminal care. The urge to carefully follow the group of (ex)cancer patients is nowadays acknowledged. However, it is still unclear what the role of PCPs can be, and to what extent this may influence the organisation of care (Hoopes *et al*. 2019). The fact that patients are living in relatively good physical condition in which the setting partly shifts from ‘clinical’ towards ‘daily life’ automatically results in different responsibilities towards health care.

In this focus group study, we explored the (1) experiences of PCPs and oncological medical specialists about providing care to patients living longer with incurable cancer and (2) their preferences concerning different care approaches (palliative support, psychological/survivorship support).

## Methods

### Design and setting

This study is part of a larger project that examines the experiences, needs and wishes of patients and healthcare professionals living longer with incurable cancer (Buiting *et al*. 2019). In this specific study, our project group further explored the collaboration between PCPs and oncological medical specialists in a focus group study, with a specific focus on the care for patients living longer with incurable cancer. In doing this, our project group ensured that all necessary items to guarantee adequate qualitative research were checked with the COREQ-checklist. This was evaluated in accordance with the standards of O’Brien *et al.* (Tong *et al*. 2007).

Because the topic of this study was relatively new to do research on, we chose to explore experiences and attitudes via different focus group sessions. A strength of focus group studies is that different participants are brought together, to which face validity increases. To facilitate the focus group discussion, we established a definitional framework beforehand (see Table 1).

**Table 1.** Operationalisation of patients living longer with incurable cancer

• A disease phase in which patients with an incurable form of cancer experience ‘stability’ or ‘remission’ as defined by their medical specialist (such as the medical oncologist). • A disease phase that lasts until the moment the patient significantly deteriorates, and the terminal phase of life approaches. • A disease phase where patients may feel rather good, and may receive anti-cancer treatment at the same time.	

### Recruitment and sampling

Our project group started three focus groups with PCPs in November 2017 (*n* = 6 PCPs) and 2 focus groups in January 2018 (*n* = 5 PCPs; *n* = 4 PCPs). When we noticed that PCPs were unaware of certain aspects in the hospital setting or felt hampered by medical specialists to have in-depth discussions about this topic, we transitioned to multidisciplinary group sessions. PCPs sometimes reported to have received information after quite a long period of time from medical specialists, through which assessing the severity of the patients’ situation became more difficult for them. In March 2018, our project group started with multidisciplinary focus groups with PCPs and medical specialists also (*n* = 9 PCPs; *n* = 2 medical specialists); May 2018 (*n* = 4 PCPs, *n* = 3 medical specialists) and June 2018 (*n* = 4 PCPs and 1 medical specialist). In the multidisciplinary group sessions, we included PCPs as well as urologists, oncologists, pulmonologists and head and neck surgeons.

All participants were recruited by snowball sampling in existing professional networks (telephone or e-mail) assisted by healthcare organisations such as the Netherlands Comprehensive Cancer Organisation (IKNL) and local organisations focusing on palliative care or oncology. Our project group took care that PCPS and medical specialists covered various experiences in every focus group. Although research members HMB and TB were present during all focus group sessions, they did not add to the discussion, apart from moderating. Our project group held three focus groups in the eastern/mid part of the Netherlands, and three focus groups in the western part of the Netherlands. Participants varied in years of work experience and gender, see also Table 2. Our project group excluded PCPs and medical specialists with less than one year of experience. Main reasons for not participating were time constraints.

**Table 2.** Respondent characteristics

	General practitioners *n* = 26	Medical specialists *N* = 6
Gender		
Male	16	2
Female	10	4

All doctors consented the focus group to be audiotaped and transcribed. Our project group checked part of the transcripts with the audio and noticed that all records were adequately described. The transcripts were anonymised to ensure the participants’ anonymity. Access to the data was limited to the researchers.

### Focus groups

The focus groups were moderated by TB (FG1, FG2, FG4) or HMB (FG3, FG5 and FG6). They were both experienced moderators. The median/mean meeting time of the focus group sessions (including breaks) was 2.5 hours. We sent all participants background information about the study in advance and received their written consent beforehand. Moreover, each focus group was started with some background oncologic information as well as a definition and clarification about patients living longer with incurable cancer. For every focus group, one meeting was held.

Subsequently, our project group presented participants with open-ended questions as well as case descriptions. We discussed four different cases that differed in treating medical specialist, type of cancer and duration of disease. As of focus group 4, our project group started a discussion about the ideal definition of the disease phase in which patients are living longer with incurable cancer and added new questions to the discussion, such as; ‘Is the role of the PCP clear to patients?’; ‘What makes chronic different from palliative?’; etc. During focus group 6, data saturation was reached as no new themes with respect to the research questions emerged. We did not use an interview guide but provided guidance via a PowerPoint presentation. In this presentation, we (again) shortly introduced the topic of patients living longer with incurable cancer, and to what extent this differs from terminally ill patients and patients that can still be cured. Moreover, we mentioned the topics which we wanted to discuss, such as familiarity with this patient group, experiences with multidisciplinary collaboration, etc.

### Data analysis

The transcripts of all six focus groups were coded and analysed using Atlas-ti 8.2. FB and HMB coded all focus groups individually. We discussed the themes and verified for interpreter consensus. We arranged several meetings to discuss themes and underlying themes/items to develop a scheme to index text fragments with similar content (in Atlas-ti 8.2). We eventually chose for 3 overarching themes: the process of awareness, the definition and marking of patients living longer with incurable cancer, and communication and caring in this disease phase. Underlying themes were for instance: the proactive role of the PCP, PCPs’ wishes, PCPs’ experiences, the care for the patient, and communication and collaboration.

By analysing the themes, through thematic analysis, hypotheses emerged and were monitored with the data.

A professional translator translated the chosen quotes that illustrated our results. The quotes are from PCPs, unless stated otherwise. According to Dutch policy, the study did not require a review by an ethics committee because the data collection was anonymous with regard to the participants (healthcare professionals) and the content of the discussions was not considered to be possibly incriminating. The consulted committee provided us with a declaration of no objection. One of the project members sent a short report to the participants straight after the focus group; we will send a Dutch version of this paper to the participants after publication. One participant also participated as a co-author in the paper by reflecting about the general findings and by editing the paper. Involving participants working in clinical practice is nowadays more often used to ensure that data will be analysed in close connection with actual medical practice (Richards *et al*. 2020).

During a period of 8 months, six focus group sessions were held with PCPs and medical specialists. In this time period, a switch in mindset seemed to have happened among PCPs. Whereas the first focus group participants initially focussed on mainstream palliative care (e.g. patients living approximately < 1 year), participants in subsequent focus groups slowly started to broaden their scope towards other patients with life expectancies of more than 1 year. Since we shared previous findings, this probably quickened the participants’ view in that the discussed patient group was somewhat different compared to the mainstream group of patients receiving palliative care (where palliative care is usually implemented if they have life expectancies of less than 6 months).

## Results

Awareness about the increasing frequency of patients living longer with incurable cancer with adequate to high quality of life, throughout the focus groups, was not only a result of the time period across the different groups. We therefore depicted this finding as one of the themes that emerged during the focus group sessions (Theme 1). Other themes were (2) the definition and recognition of a disease phase in which patients are living longer with incurable cancer, and (3) communication and caring in this disease phase.

### Awareness of patients living longer with incurable cancer

In the first focus group, most PCPs reported that they considered patients living longer with incurable cancer as similar to the palliative disease phase (which, in general, concerns a patient with a life expectancy of ~ 1–6 months). They described timely marking of the palliative phase as crucial for appropriate care, referring to ongoing projects such as PATZ (a project stimulating palliative home care) (van der Plas *et al*. 2014). PCPs reported to follow the ‘surprise question’ if answered negatively (e.g. ‘Would you be surprised if the patient died within the next 12 months?’) as the starting point of initiating palliative care. During the first focus group, participants only spoke about patients with an estimated life expectancy of less than one year, as defined by the common palliative care approach.

Interestingly, in our third, fourth and fifth focus groups, the disease trajectory we were focusing on seemed to be more accepted as a distinct part of the patient’s disease trajectory (e.g., separate from the terminal disease stage). Both PCPs and medical specialists did not associate these patients with having a terminal form of cancer and/or an approaching death although they were aware of the incurable nature of their disease. The difficulties they described in defining this specific disease phase, e.g., patients living longer with incurable cancer, were partly because they considered this to be a grey zone.

Box 1. *Respondent 2: Well, anyway, we were talking about the intermediate phase [patients do not request palliative care specifically, but are aware of the incurable nature of the disease].This is quite a different phase.*

*[Focus group 5]*

Although PCPs acknowledged their added value in this specific disease phase, they also noted that their level of involvement should depend on the patient’s preference. Some reported to be aware of the fact that if patients preferred to stay in touch with their PCP, the chance of receiving further treatment could be lower than when they (also) preferred to stay in contact with their medical specialist.

Box 2. *Put it another way, if you are going to ask for advice, you search to see who you want to ask. And you know that if you go to the medical specialist, you’ll get suggestions for further treatment and if you go to your PCP, you will get a more palliative approach, people know that… [Focus group 5]*

Medical specialists agreed that patients living longer with incurable cancer should be approached differently than patients in the palliative phase of cancer (e.g. estimated life expectancy < 1 year). They acknowledged this disease trajectory as a distinct phase compared to mainstream palliative care. Interestingly, instead of PCPs’ problems regarding the dichotomy ‘incurable/mainstream’ palliative care (< one year to live), medical specialists especially focused on the dichotomy ‘curable/incurable’.

Box 3. *Respondent (medical specialist): For me, as a medical specialist, of course my preference is to be on time, if anything can still be treated, be cured. […] So I monitor someone closely if possible, or if necessary, whereas if you know someone can’t be cured any more, then the focus is on the quality of life and prolonging life if possible. Then you have a very different attitude.*

*[Focus group 4]*

### Defining and differentiating different disease phases

During all group sessions, the participants did not always speak about the same disease phase, despite our efforts to clearly define this disease phase beforehand. Participants sometimes used the terms protracted incurable cancer, the terminal disease phase and the palliative disease phase interchangeably.

Insecurity about prognosis could result in miscommunication surrounding terminology: During the sessions, there seemed to be no consensus or shared definition between healthcare professionals on patients living longer with incurable cancer. Accordingly, most participants experienced problems in providing a specific name to this disease phase. Whereas some of the PCPs and medical specialists reported that ‘chronic disease’ did not fit with the disease trajectory of these patients, others were very much in favour of this term.

Box 4. *Respondent 1: Right, ‘chronic’ doesn’t really fit but on the other hand there’s no better term for it. […]*

*Respondent 2: Right, diabetes is a chronic condition but you don’t die from diabetes, you die from the complications. That’s the difference with chronicity.*

*Respondent 1: So, what is a chronic disorder?*

*Respondent 2: Diabetes is a chronic disorder.*

*Respondent 3: Yes, but what’s the definition of a chronic disorder?*

*Respondent 2: Something you’re stuck with for the rest of your life.*

*[…]*

*Respondent 2: Why do you actually have to define […]*

*Respondent 3: It’s handy to define something we are all talking about. [Focus group 4]*

The major problem both PCPs and medical specialists addressed was that care among patients living longer with incurable cancer could only be distinguished from the terminal/mainstream palliative disease phase *in hindsight*. Most of the participants considered ‘chronic’ a correct term.

Box 5. *Respondent 1: Can you just look back afterwards or not? Can you already kind of say, “Well, this will be a chronic phase”?*

*Respondent 2: (medical specialist) Yes, that’s all quite tricky… yes, pretty awkward.*

*Respondent 3: Because when do you decide that? If a therapy starts to work or whatever after the first phase, do you then say “Now it’s stable”?*

*Respondent 2: (medical specialist) Right, then I say “It’s stable now, you know”. Our policy is to wait and see, then someone comes to the outpatient clinic a few months later. I don’t say then, “I hope an intermediate phase is starting but equally it could go wrong in three months”.*

*[Focus group 5]*

PCPs generally acknowledged their role in assisting patients in the very last stage of life. They however reported to prefer to contact patients in an earlier stage of their disease as well. Some of the medical specialists reported to try to contact the PCP by phone when the patients’ condition deteriorated, in line with the preferences of most PCPs in this study. ‘We need each other’ was a frequent comment. Medical specialists worried that contacting all PCPs about this expanding patient group, on time, will become unfeasible in future. Other problems that medical specialists themselves faced were time constraints, the struggle in finding the appropriate care role, a lack of knowledge on and experience with this specific disease phase and dealing with an uncertain prognosis.

Box 6. *Medical specialist: Well, then you get, um… you get that drug A. 70% chance of you still being around one year on. It works out fine. A year later, you see a recurrence, but you don’t take it too hard: ah, resistance. “Oh well, I’ve still got another drug”. Again it’s a 60% chance, but this also works out fine. Well, anyway, you carry on like this but it’s really the same every time: You go along, you tell them again that they’re dying as it were, the progression… then a couple of weeks later you say “No, I’ve got something for you”. I’m really pleased. […] How do you cope emotionally, how can you manage? [Focus group 4]*

### Communication and care in a trajectory of patients living longer with incurable cancer

The PCPs’ main concern was to guarantee optimal communication with patients as well as medical specialists. They often reported barriers when they tried to reach medical specialists. Accordingly, being able to approach their patients on time if medical specialists did not contact the PCPs themselves seemed difficult. Moreover, participants were hesitant to reach out to their patients. They doubted whether patients in fact desired additional contact with their PCPs on top of the contact with their medical specialist. As a result, many PCPs decided to adopt a ‘passive’ attitude in this disease phase, for example, awaiting whether the patient would approach them, although they themselves were willing to see them.

Box 7. *Well, the first thing that I find remarkable [reading this case scenario] is that the patient is saying ‘I do not need any extra care from the PCP’. As* a PCP, I’d actually quite like to know, really find out from such a p*atient what the developments with respect to her cancer are, so I like to see those patients from time to time to discuss that. [Focus group 1]*

This passive attitude of PCPs was partly related to the fact that these patients were regarded as patients in good condition, for example, the ‘chronic’ cancer patients. PCPs mostly explored what was going on in the patient’s life – regarding work, relations and their mental and physical condition (if patients requested a consultation with their PCP). PCPs with special education in palliative care felt they generally were more engaged with patients with cancer than colleagues who had not followed this course/had no special knowledge about palliative care; they seemed to be more inclined to contact patients themselves. Nevertheless, all of them reported difficulties in tracing these patients at the right time and finding the time to contact these patients if they did not contact the PCPs themselves.

Box 8. *Yes, but what’s tricky is how to keep an eye on everyone. We’ve got a big practice with just the two of us, 4500 patients and that’s quite a lot of people.*

*Then I say “I’ll do it (taking care for a patient living longer with incurable cancer)” but occasionally I think “Oh no, I completely forgot”… so that’s… you want to (taking care/monitoring these patients) but I can’t; the way I do things at the moment, I can’t keep track of everyone. [Focus group 3]*

Some patients asked their PCPs for a ‘second opinion’. After having heard the advice of their medical specialist, they discussed their options with their PCP. A large proportion of PCPs reported they felt somewhat incompetent for this task because they were not up-to-date regarding the latest developments concerning anti-cancer treatment. PCPs reported to highly appreciate easy-to-understand letters from medical specialists when treatment decisions had been made or serious side-effects emerged.

Box 9. *Respondent 1: So if they say, “I’d rather get it from the PCP”… I’ve also had people saying, “Well, I heard this story from the specialist; now I’m coming to you to discuss this a bit”…*

*Respondent 2: Yes, exactly.*

*Respondent 1: But then of course you need to be informed as a PCP about what they said so that you can respond properly, because PCP can’t keep up with all the new techniques and studies that are going on.*

*[Focus group 1]*

## Discussion

### Statement of principal findings

In this focus group study, we consecutively held three group discussions with PCPs and three multidisciplinary group discussions of PCPs and medical specialists about patients living longer with incurable cancer. PCPs as well as medical specialists acknowledged that providing care to these patients is both challenging (e.g. patients were living longer) and complex (due to the unpredictable disease trajectory, prognosis and side-effects). They also struggled in finding the right label for this specific disease phase, using the terms ‘stable’, ‘chronic’ and ‘palliative’ interchangeably. All participants acknowledged problems in the communication, both with patients and colleagues. Whereas PCPs generally preferred a proactive role with their patients, they reportedly stayed passive in some cases, leaving the initiative of PCP consultation to the patient.

### Strengths and weaknesses of the study

This study explored a relatively new topic and illustrated an important hiatus in (primary) oncologic care. Although the number of patients living longer with incurable cancer is growing, many PCPs did not acknowledge this disease phase as different compared to the terminal disease phase (<6 months life expectancy). Although this could be considered a limitation of the study, it was also an important finding. Furthermore, we evaluated how opinions and ideas developed throughout the study period. A feature that increases the validity of this study is the multidisciplinary composition of the focus groups, in which ideas and information were exchanged between PCPs and medical specialists. A great advantage is the immediate exchange of ideas and information when something may appear to be unclear by one of the focus group members. We specifically chose to the diverse group composition to be able to discuss this topic in the most broadest sense (e.g. young/old, palliative/not palliative minded, male/female, etc.). Our study has limitations too. First, recruiting participants depending on the use of local contacts is susceptible to ‘volunteer bias’. Since we recruited participants via various organisations and persons we believe that this form of bias is limited. Second, one of the focus groups was rather small (*N* = 4). Third, social desirable answers might have been given, especially since some participants were acquainted. Fourth, in our first three focus group sessions only PCPs participated. It might have been better to start with multidisciplinary groups since the interaction between PCPs and medical specialists significantly improved the outcomes of the discussions. Fifth, focus groups are susceptible to moderator bias. We however followed a standard scheme and did not try to incorporate our own opinion in any way. Finally, we did not include the patient perspective in this study and our findings cannot be transferred to all contexts, which is interesting to explore in future studies.

### Findings in relation to other studies

#### Defining the trajectory of patients living longer with incurable cancer

Awareness of patients living longer with incurable cancer than the estimated period of one year (the recommended period for ‘mainstream’ palliative care) is the first step to improve care for these patients (Boyd *et al*. 2019; Buiting and Bolt 2019; Schildmann *et al*. 2020). Our study convincingly showed that even in those 8 months study period, a switch in mindset seemed to have arisen. Apart from awareness about this disease phase due to media attention, more clarity about the impact of choosing specific labels for this disease phase on patients’ well-being (and accordingly their decision-making capacity) is another important step. Also, because labels may influences how healthcare professionals themselves act. Most of our participants were unsure what the appropriate disease label for this specific disease phase should be. It however could be argued whether one specific disease label would be worthwhile for this disease phase, and/or whether labels to a certain extent differ between different stakeholders (e.g. healthcare professional, patient, policymaker, etc.). It is probable that a disease label that is used among healthcare professionals (across different healthcare professionals/in the medical record) and towards patients (during consultations) can have important implications. At first sight, the disease label that is chosen seems a strong fundamental in formulating treatment aims (doctor perspective) as well as coping strategies, well-being and treatment decisions (patient perspective), which to a certain extent differs across disciplines.

Our study for instance showed that medical specialists are more inclined to differentiate between the labels curable/incurable instead of incurable/the last stage of life. This does not mean that they also communicate this as such towards their patients. In fact, in the very last stage of life (life expectancy of a couple of months) it is generally the PCP and not the medical specialist taking care for the patient, which could be one explanation for this difference in mindset. Moreover, the difference could also be explained by the fact that medical specialists take care for their patients while providing treatment, whereas PCPS have in particular a supportive role (if consulted) about the patient’s life-story. Although it is generally agreed that medical specialists need to prevent overtreatment (Buiting *et al.* 2011; van Ommen-Nijhof and Sonke 2022; The *et al.* 2000), it at the same time seems logical that medical specialists have a different mindset and are inclined to advise differently than PCPs while they know their patients’ treatment trajectory for such a long time (Buiting *et al.* 2019). They probably can better estimate whether additional treatment could be beneficial or not.

#### Knowledge

Both PCPs and medical specialists reported a lack of knowledge on patients living longer with incurable cancer. This is not surprising, since this phenomenon is new for many physicians (Buiting *et al*. 2019). Current literature on survivorship care rarely specifically touches upon patients with incurable cancer (Vijayvergia *et al*. 2015), while literature in palliative care generally excludes patients living with a metastasised form of cancer, for more than one year. However, the number of studies that describe patients living longer with incurable cancer slowly increases in for instance breast cancer, lung cancer and prostate cancer (Buiting *et al.* 2019; Harley *et al.* 2015). It thus seems that patients both receiving anti-cancer treatment and (if wanted) supportive/survivorship care currently mainly rely on the oncologist, for example, anti-cancer treatment either in combination with paramedical care. With the introduction of a new journal, for example, BMJ Palliative & Supportive Care, attention and overlap towards both disciplines seem to increase. Still, supportive care is primarily focused on patients who can be cured, whereas palliative care is primarily focused on patients in their last year of life. Using different terminology to a great extent seems to determine how care is circumvented, for example, for patients hearing about stage IV disease (medical jargon for metastasised disease) is different compared to patients with an incurable form of cancer.

Today, new treatment options, such as immunotherapy and checkpoint inhibitors, can have astonishing effects, with longer survival rates and lower risk of side-effects (Blank *et al*. 2016). At the same time, new side-effects are observed, which are to a great extent unknown to both PCPs and medical specialists. It is therefore not unexpected that participants were more or less uncertain about the effects of these new anti-cancer drugs, and accordingly about treating patients in this specific disease phase. Preventing reluctance of PCPs wanting to become involved, a clear role of both specialties need to be further explored. We previously reported that – at present – the role of PCPs taking care for patients living longer with incurable cancer is mostly limited to the psychosocial aspects of the decision-making process and treatment of common comorbidities (Buiting and Bolt 2019).

A study of Klabunde *et al* reported barriers to effective communication between PCPs and medical specialists in survivorship care (curable and incurable) (Klabunde *et al*. 2013; Klabunde *et al*. 2017). Bringing expertise and experiences together and weighing up the available options could possibly improve the decision-making process. Combining the strengths of the medical oncologist (adequate provision of anti-cancer treatment, doctor-patient communication) and the PCP regarding oncology patients (supportive care, doctor-patient communication, life course medicine) may in certain situations be ideal. Integrating elements of shared care in a multidisciplinary setting is challenging and more research in this field is warranted. It requires a comprehensive and multidisciplinary care infrastructure between various healthcare professionals (Doull 2012; Loonen *et al*. 2018).

### Conclusions for research and/or practice

Providing care to patients living longer with incurable cancer (e.g., a life expectancy of at least 1 year) is considered both challenging (e.g. patients were living longer) and complex (due to the unpredictable disease trajectory, prognosis and side-effects). Using specific labels towards patients (next to other elements that determine patients’ well-being during consultations (Buiting *et al.* 2020, 2022)) can have a tremendous impact on patients’ well-being, and accordingly, which decisions they for instance would like to make regarding anti-cancer treatment. Both PCPs as well as medical specialists need to be aware of using terms such as ‘stable’, ‘chronic’ and ‘palliative’ interchangably (Buiting *et al.* 2023). Although this exploratory research provides indications that the term ‘chronic’ would suit patients in this disease phase best, our research in which the patient perspective is included also should strengthen these results even more.

- PCPs will have an increasing number of patients living longer with incurable cancer in their practice. However, in a single PCP practice, the experience with incurable cancer patients remains low, partly because patients often prefer to stay in contact with their specialist. PCPs as well as medical specialists are unsure how we should label these patients best, and how their care can be guaranteed. The development of an education module could possibly add in motivating PCPs and medical specialists to find options to better interact with each other and to make better demarcations between the ‘mainstream’ palliative disease phase and longer disease phases with metastatic cancer.
- Medical specialists in particular will be more aware of the group of patients living longer with incurable cancer, including their care needs while receiving anti-cancer treatment. They can have an important role by timely referring their patients to their PCPs. During the total period of patients living longer with incurable cancer, contact with both PCPs and medical specialists seems preferable.
